# A Novel Prognosis Signature Based on Ferroptosis-Related Gene DNA Methylation Data for Lung Squamous Cell Carcinoma

**DOI:** 10.1155/2022/9103259

**Published:** 2022-09-12

**Authors:** Xuequan Wang, Yinnan Meng, Changxin Liu, Haihua Yang, Suna Zhou

**Affiliations:** ^1^Key Laboratory of Radiation Oncology of Taizhou, Radiation Oncology Institute of Enze Medical Health Academy, Department of Radiation Oncology, Taizhou Hospital Affiliated to Wenzhou Medical University, Taizhou, Zhejiang Province 317000, China; ^2^Department of Epidemiology and Biostatistics, School of Public Health, Xi'an Jiaotong University Health Science Center, Xi'an, Shaanxi 710061, China; ^3^Department of Radiation Oncology, Xi'an No. 3 Hospital, The Affiliated Hospital of Northwest University, Xi'an, Shaanxi 710018, China

## Abstract

Ferroptosis-related genes regulating an iron- and lipid reactive oxygen species (ROS)-dependent form of programmed cell death suggest critical roles for ferroptosis in cancers. However, the prognostic value of ferroptosis-related epigenetic features such as DNA methylation in lung squamous cell carcinoma (LUSC) needs to be studied. Ferroptosis-related genes are collected from the FerrDb database, and the methylation data of these related genes in LUSC methylation data downloaded from the TCGA are retrieved. The DNA methylation data (362 LUSC samples) were analyzed to screen prognostic ferroptosis-related methylation sites. After patients with complete overall survival (OS) information were randomly separated into training cohort (*n* = 200) and validation cohort (*n* = 162), the least absolute shrinkage and selection operator (LASSO) and the Cox regression were used to establish and validate the prognostic signature. The time-dependent receiver operating characteristic (ROC) and Kaplan–Meier survival curve analyses, Harrell's concordance index (*C*-index), calibration analysis, and decision curve analysis (DCA) were performed to evaluate the risk signature and related nomogram. A series of other bioinformatics approaches such as mexpress, cbioportal, maftools, string, metascape, TIMER, and Kaplan–Meier survival curve analysis were also used to determine the methylation, mutation status, protein interaction network or functional enrichment, effects on immune cell infiltration, or expression level prognosis of those signature-related genes. A total of 137 DNA methylation sites were identified as prognostic predictors corresponding to 109 ferroptosis-related genes (FRGs). The methylation signature containing 31 methylation sites proved to be superior predictive efficiency in predicting the 1-, 3-, 5-, and 10-year OS. 8 out of 28 signature-related genes were significantly related to OS time or OS state in patients with LUSC. In addition, DUSP1, ZFN36, and ALOX5 methylation status also correlated with pathological *M* and ALOX5 methylation correlated with pathological *N*. The prognostic prediction efficiency of *T*, *N*, *M*, and the stage was inferior to that of the DNA methylation signature. LUSC patients in the high-risk group own a significantly larger number of variants of FRGs than those in the low-risk group. In addition, negative or positive correlation patterns were presented among the different infiltrating immune cells with risk scores or signature-related genes in patients with LUSC. The expression level of 15 signature-related genes showed a significant relationship with OS of LUSC patients. A novel prognostic nomogram survival model containing 4 factors including age, pathologic *T*, stage, and risk group was constructed and validated, And*C*-index, decision curve analysis (DCA), and calibration analysis demonstrated its excellent predictive performance. The FRG DNA methylation data-based prognostic model acts as a powerful prognostic prediction indicator in LUSC patients and is advantageous over the traditional model based on *T*, *N*, *M*, and stage.

## 1. Introduction

Lung cancer is a common malignancy worldwide with high morbidity and mortality [1]. Non-small-cell lung cancer (NSCLC) accounts for approximately 85% of all lung cancer patients, and LUSC accounts for 20–30% of NSCLC cases [2, 3]. The 5-year OS was only 4–17%, which was associated with late diagnosis, a high rate of postoperative recurrence, and resistance to the multimodal intervention including chemotherapy, radiation therapy, and targeted therapy [4]. The median survival time of LUSC patients was approximately 30% shorter than nonsquamous NSCLC [5]. The specific clinicopathologic characteristics of LUSC, such as advanced stage at diagnosis, older age, central tumor location, and comorbidities, contribute to the poorer prognosis [6]. There are limited options and benefit in the treatment for patients with LUSC in comparison with the successful advance of the targeted therapy in lung adenocarcinoma. To screen out the LUSC patients with the higher risk for poor prognosis and then to analyze the high-risk factors or gene-phenotype may be helpful to develop new powerful targeted therapy for LUSC cases. However, the traditional prognostic prediction depends mainly on the histopathologic type and tumor/node/metastasis (TNM) staging system and is not effective for accurately predicting the outcomes of LUSC patients. Therefore, it is urgent to establish a precise prognosis predictive model to distinguish high-risk groups of cancer-related death in LUSC.

Ferroptosis is a newly recognized type of programmed cell death characterized by the accumulation of iron-dependent lipid hydroperoxides [7]. Ferroptosis has been confirmed to inhibit tumor progression and plays an important role in various human cancers including lung cancer [8–10]. A series of strategies including FDA-approved drugs, chemical compounds, and genes have been developed to induce ferroptosis of cancer cells, prefiguring that targeting ferroptosis may become a novel promising antitumor treatment [8]. Many FRGs are expressed at an abnormal level in tumors. However, the role of ferroptosis and FRGs are still not fully studied in LUSC. Recently, genes identified from FRGs were applied to construct various gene signatures that predict the prognosis of hepatocellular carcinoma, lung adenocarcinoma, and bladder cancer patients in the cancer genome atlas (TCGA) cohort [11–13]. Although Diao et al. constructed a LUSC prognostic model with 16 FRGs, the maximum value of roc was only 0.71 for OS prediction [14]. Thus, it is necessary to find a more accurate prognostic model based on FRGs. LUSC with a poor prognosis is more common in smokers and elderly people, which affects DNA methylation. DNA methylation is an important epigenetic change involved in the regulation of gene expression without any changes in the basic nucleotide sequence in numerous biological processes [15]. Abnormal DNA methylation occurs in tumorigenesis, and several methylation biomarkers have been used to predict prognosis in LUSC [16–18]. DNA methylation-based signatures can provide an effective prognosis and identify potential cancer treatments. At the International Association for the Study of Lung Cancer (IASLC) 2021 World Conference on Lung Cancer (WCLC), we reported the preliminary result as a format of abstract, which suggested a prognostic model based on FRGs methylation sites had a more accurate prediction of OS. In this study, we intend to publish detailed and further data about our model contributing to researchers a more systematic and comprehensive understanding of our prognostic model.

## 2. Materials and Methods

### 2.1. Data of LUSC Cases from TCGA

The DNA methylation data of LUSC based on the Illumina Human Methylation 27 platform were downloaded from the TCGA database (https://cancergenome.nih.gov/) according to the following criteria: (i) available histological data that could determine squamous cell carcinoma; (ii) each case had complete prognosis information; and iii) other clinical information needed was available. A total of 362 LUSC cases with complete OS information, RNA sequencing data, and methylation data from TCGA were selected for further analysis. We also downloaded the raw RNA-seq data, prognostic data, clinical features, and information about aberrantly methylated sites. The 258 genes related to ferroptosis, including drivers, suppressors, and markers, were collected from the FerrDb database (https://www.zhounan.org/ferrdb/) in Legacy version [19]. The quality control of the intensity data and aberrantly methylated sites of FRGs were calculated by “ChAMP” package (https://www.bioconductor.org/packages/release/bioc/html/ChAMP.html). The genomic coordinates of the methylation site of FRGs were gained depending on GRCh38. *R* version 4.03 was applied to data analysis.

### 2.2. Prognostic FRG Methylation Site Identification and FRG Signature Construction

Prognostic methylation sites were firstly screened by univariate proportional hazard analysis for FRGs methylation sites, with a *p*-value of less than 0.05 as a significant difference cut off value based on the methylation data of 362 LUSC samples. Then, the LUSC cases were randomly separated into a training cohort (*n* = 200) and a validation cohort (*n* = 162) by “caret” package. Lasso regression was performed for the training cohort by the “glmnet” *R* package to eliminate collinear or correlated methylation sites and the qualified sites were selected based on the minimum mean cross-validated error. In addition, the risk score was calculated according to the following formula (*n* represents the number of signature-related methylation sites, Meth_*i*_ represents the methylation *β* value of each gene, and *β*i represents the coefficient index, respectively):

(1) $$\begin{array}{c} \text{Riskscore}=\sum_{i=1}^{n}\left({\text{Meth}}_{i}*\beta_{i}\right). \end{array}$$

### 2.3. Identification Prognostic Methylation Sites of FRGs

The methylation sites of FRGs were grouped into the low- and high-risk scores by the survminer package as described before [20]. Kaplan–Meier survival analysis was further established to analyze the OS difference between the hypermethylated and hypomethylated groups of each model-associated methylation site. Moreover, MEXPRESS was utilized for presenting the expression, DNA methylation, and clinical parameters of those model-related genes (https://mexpress.be) [21].

### 2.4. Gene Functional Enrichment Analysis and PPI Network Analysis of Core FRGs

Metascape (https://metascape.org) [22] was employed to gain insights into the biological functions of 28 prognostic model-related FRGs. By filtering with the criterion of a *p* value <0.01, minimum count of 3, and an enrichment factor >1.5, the target genes of these FRGs were selected and classified into clusters depended on their biological functional similarities. The interrelation of 28 FRGs was analyzed by the Search Tool for the Retrieval of Interacting Genes (STRING) database (https://string-db.org) [23] to conduct the protein-protein interaction (PPI) enrichment network with the confidence score of 0.4.

### 2.5. Prognosis and Mutations of Genes Involved in the FRGs Signature

Oncoplot function in “maftools” *R* package was applied to generate waterfall plots of FRGs mutation in LUSC patients with a low- and high-risk score of FRG methylation sites [24], respectively, which presented mutation classification, mutation frequency, and single-nucleotide variant (SNV) class frequency for LUSC samples. We also plotted the variant allele frequency (VAF) distribution of FRGs. Kaplan–Meier (KM) survival analysis with the log-rank test was utilized to analyze the OS difference among LUSC patients with unaltered model-related FRGs using cBioPortal bioinformatics tools (https://www.cbiop/ortal.org/). A univariate Cox regression analysis was performed to compare the risk probability of FRGs mutation between LUSC patients with a low- and high-risk score of FRG methylation sites.

### 2.6. Immune Infiltration Analysis

The levels of infiltrating immune cells such as T lymphocytes and natural killer(NK) cells of LUSC were quantified by the CIBERSORT method [25]. Finally, the Pearson correlation coefficient to analyze the coefficient of correlation between FRGs-related prognostic risk score and infiltrating immune cells was used. Then, a heatmap was used to display these results.

### 2.7. Construction and Test of the Nomogram

The LASSO Cox regression analysis was used to further screen the prognostic factors in the methylation risk score group with clinicopathological parameters (*T*, *M*, *N*, age, and stage). A nomogram was constructed by the “rms” *R* package [26] based on the factors with *p* ≤ 0.05 from multivariate Cox proportional hazard analysis. The predictive and discriminative ability of the nomogram for 1-, 3-, 5-, and 10-year OS was weighed using ROC, Harrell's concordance index (*C*-index) via the “survival” *R* package, and calibration plots via Hosmer–Lemeshow test. In addition, the nomogram was tested by proportional hazard assumption to analyze that whether the risk score included in our model was independent prognostic indicators. To evaluate the accuracy and effectiveness of our model, 7 prognostic models of LUSC from published papers [17, 18, 27–31] were reviewed and studied with a comparison. Net reclassification index (NRI) and integrated discrimination improvement (IDI) are the common algorithms used to assess the model in recent years [32, 33]. Therefore, we applied the NRI and IDI methods of the “survIDINRI” package to compare our model with the above model. The *p* value <0.05 was statistically significance.

### 2.8. Validation of the Multi-FRG Methylation Site-Related Prognostic Signature and Nomogram

First, the Kaplan–Meier curve with the log-rank test was performed to evaluate the OS difference between the high-risk and low-risk groups combined with the clinical stage. To evaluate the risk model and the nomogram, we used “timeROC” [34], “rmda”(https://mdbrown.github.io/rmda/), and “survcomp” [35] packages to conduct the ROC curve analysis, *C*-index, calibration analysis, and decision curve analysis.

### 2.9. Statistical Analysis

The Kruskal–Wallis chi-squared and independent *t*-test were used to compare the difference between two groups. *R* package “survival” was performed to conduct univariate and multivariate Cox regression analysis along with hazard ratios (HRs) and 95% confidence intervals (CIs). The Pearson correlation coefficient was applied to analyze the coefficient of correlation among variables. *p* < 0.05 or *p* < 0.001 was identified as statistical significance according to specific requirements.

## 3. Result

### 3.1. Characterizing a Large Methylation Heterogeneity in LUSC Tumor Tissues

The LUSC Illumina 450K DNA methylation data of 412 samples from TCGA contain data from 372 tumor samples and 40 paired tumor samples. The chip analysis methylation pipeline (ChAMP), a Bioconductor *R* package, was used for the analysis of the methylation data [36]. Then, the density and multidimensional scaling (MDS) of all LUSC normal and tumor or normal and paired tumor methylation were analyzed. As shown in Supplementary Figure 1, methylation levels of tumor samples show more significant individual heterogeneity compared with normal samples in both the total samples and paired samples (Supplementary Figures 1(a), 1(b), 1(d), and 1(e)). Sample clustering also differed between the two groups, and normal samples have short cluster distances (Supplementary Figures 1(c) and 1(f)). After normalization, PCA on the methylation levels of 4365 FGR methylation sites showed that these FGRs methylation sites could mostly divide the samples into normal and tumor groups (Supplementary Figures 1(g) and 1(h)).

### 3.2. Identification of DNA Methylation Signature of FRGs Associated with Prognosis

**Figure 1:**
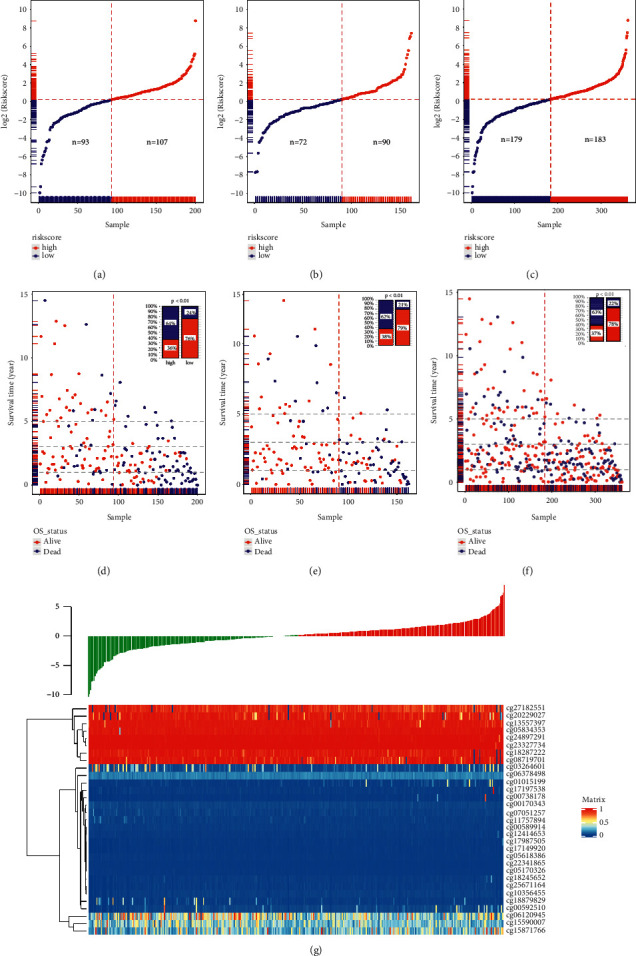
The prognostic risk score correlates OS for TCGA LUSC patients. (a)–(c) The risk score distribution was sorted from the largest to the smallest of the low-risk group and high-risk group in the training or validating or total group. Blue dots indicate the low-risk group and red dots indicate the high-risk group. (d)–(f) With increasing in the risk score, the number of dead patients increased in the training or validating or total cohort. The percentage of alive (red) and dead (blue) is marked at the top right. (g) The heatmap of 31 risk signature-related methylation sites' methylation level.

Univariate Cox proportional hazards regression model was used to evaluate the effect of the DNA methylation sites of FRGs on prognosis. The 137 DNA methylation sites were identified as prognostic predictors including 53 favorite and 84 harmful sites by univariate Cox proportional hazards regression (Supplementary Figure 2(a)). These 137 prognostic predictors corresponded to 109 FRGs. To further evaluate the prognostic value of the OS significance-related methylation sites, LASSO Cox regression analysis with 10-foldcross-validation was performed. Finally, 31 methylation sites of 28 FRGs, including cg24897291, cg11757894, cg00170343, cg06120945, cg18287222, cg01015199, cg18245652, cg05618386, cg17987505, cg27182551, cg13557397, cg25671164, cg18879829, cg22341865, cg17149920, cg17197538, cg00592510, cg15590007, cg00589914, cg20229027, cg05834353, cg00738178, cg07051257, cg08719701, cg23327734, cg12414653, cg03264601, cg06378498, cg05170326, cg10356455, and cg15871766 related to OS with nonzero coefficients were selected as a linear model candidate predictors (Supplementary Figures 2(b) and 2(c)). After independent predictors were identified and their coefficients were determined, the risk score was developed as the following formula: risk score = (−10.896 *∗* cg24897291 + −19.955 *∗* cg11757894 + −22.108 *∗* cg00170343 + −1.335 *∗* cg06120945 + −2.4426 *∗* cg18287222 + 4.2699 *∗* cg01015199 + 9.9509 *∗* cg18245652 + −82.288 *∗* cg05618386 + 50.749 *∗* cg17987505 + −2.0942 *∗* cg27182551 + −3.0395 *∗* cg13557397 + 30.497 *∗* cg25671164 + −3.0011 *∗* cg18879829 + −158.35 *∗* cg22341865 + 95.053 *∗* cg17149920 + 3.1434 *∗* cg17197538 + 2.8088 *∗* cg00592510 + −1.7002 *∗* cg15590007 + −74.871 *∗* cg00589914 + −2.1974 *∗* cg20229027 + 8.2263 *∗* cg05834353 + 5.5045 *∗* cg00738178 + −15.809 *∗* cg07051257 + −1.9767 *∗* cg08719701 + −13.756 *∗* cg23327734 + 35.481 *∗* cg12414653 + −1.5625 *∗* cg03264601 + 5.7681 *∗* cg06378498 + −65.98 *∗* cg05170326 + 24.237 *∗* cg10356455 + 0.66937 *∗* cg15871766). The risk score was then used for stratifying LUSC patients into high-risk and low-risk groups at the best separation cutoff of the risk score, by the “Surv_cutpoint” function in “survminer” package (Supplementary Figure 2(d)). There was no difference in clinical characteristics between training and validating set (*p* < 0.05) (Supplementary Table 1). The risk score distribution and related OS information for those training or validating group patients are shown in Figure 1. As the risk score increased, the number of deaths gradually increased and the survival time gradually decreased. The risk scores of patients were closely connected with the survival rate. 63% of patients in the high-risk group died compared to 22% in the low-risk group in the total cohort (Figure 1(f)). Heatmap of the methylation signature-related sites was grouped by risk score as shown in Figure 1(g). Among these 31 methylation sites, there are 8 hypermethylated sites and 20 hypomethylated sites in lung squamous cell carcinoma.

**Figure 2:**
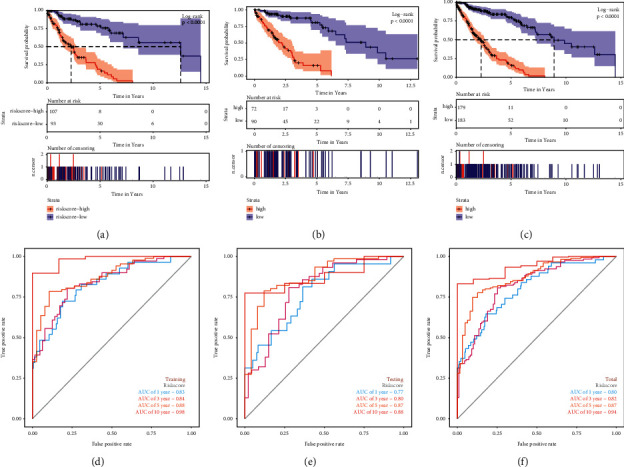
Prognostic evaluation of FRGs signature in LUSC patients. The Kaplan–Meier survival curve of the high- and low-risk groups in (a) the training cohort; (b) the validating cohort; (c) the total cohort. Time-dependent ROC analysis for predicting the 1-, 3-, 5-, and 10-year overall survival of LUSC patients in (d) the training cohort; (e) the validating cohort; (f) the total cohort.

The survival analysis was performed in the training, validating, and total cohorts to investigate the prognostic significance of the DNA methylation signature of FRGs by using the Kaplan–Meier method. As a result, patients in high-risk groups presented a significantly shorter overall survival than low-risk groups in the training cohort (HR 8.33, 95% CI 4.42–15.79, *p* < 0.001, Figure 2(a)). Meanwhile, the same results were found in the validating cohort (HR 6.67, 95% CI 3.92–11.29, *p* < 0.001, Figure 2(b)) and the total cohort (HR 7.24, 95% CI 4.83–10.78, *p* < 0.001, Figure 2(c)). In the total cohort, the 1-, 3-, 5-, and 10- year survival rates for the high-risk group were 74.2%, 35.0%, 17.2% and 0, and for the low-risk group were 95.3%, 86.1%, 79.6% and 44.9%, respectively. The ROC analysis was used to determine the sensitivity and specificity for the DNA methylation signature of FRGs in predicting the 1-, 3-, 5-, and 10-year OS. The AUC for 1-, 3-, 5-, and 10-year OS was 0.83, 0.84, 0.88, and 0.98 in the training cohort (Figure 2(d)), 0.77, 0.80, 0.87, and 0.88 in the validating cohort (Figure 2(e)), respectively, and were all above 0.80 in the training and the total cohort (Figure 2(f)) indicating that our FGRs-based methylation signature had superior predictive efficiency.

### 3.3. Comparison of the Prognostic Prediction Efficiency of DNA Methylation Signature with Clinical Predictors

**Figure 3:**
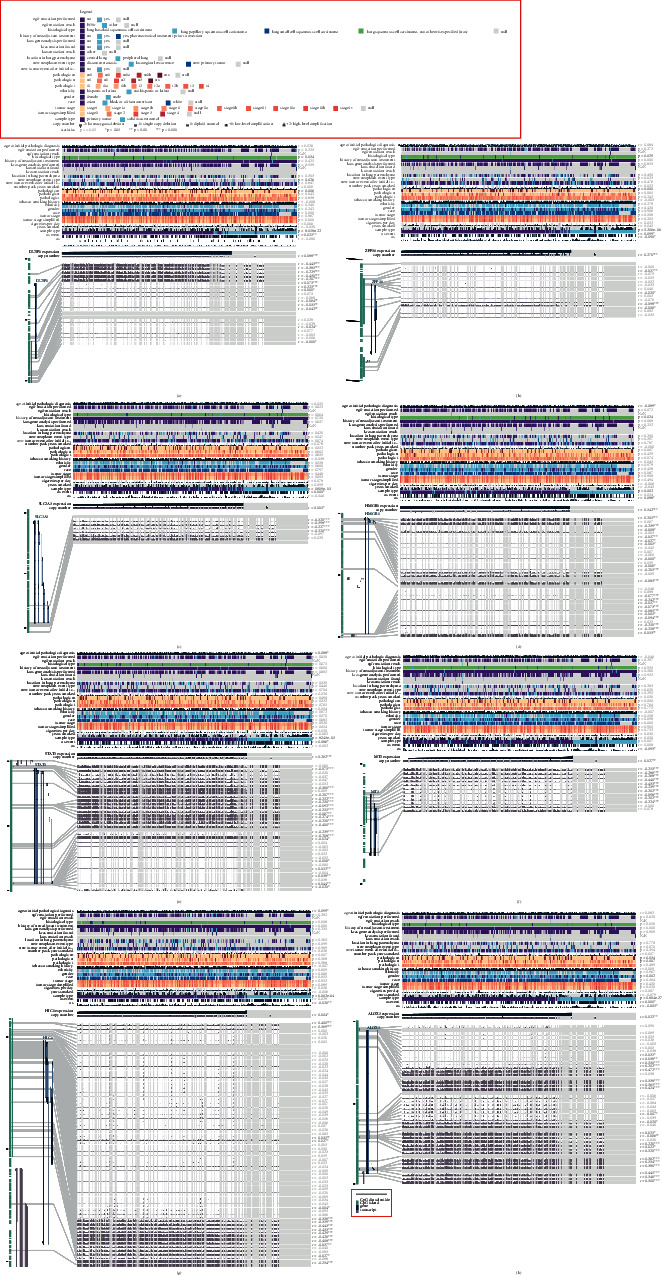
MEXPRESS applied to analyze the DNA methylation level of 29 signature-related genes. The methylation level of the probes with different clinicopathological features for those genes whose methylation level showed a significant relationship with overall survival time or overall survival state in patients with LUSC is depicted: (a) DUSP1, (b) ZFP36, (c) SLC2A3, (d) HMGB1, (e) STAT3, (f) MT3, (g) HIC1, and (h) ALOX5.

The methylation level of 28 signature-related FRGs and the pathological features of LUSC were evaluated by the MEXPRESS website (https://mexpress.be/). Among 28 FRGs, the overall DNA methylation status of dual-specificityphosphatase-1 (DUSP1), zinc finger protein 36(ZFP36), solute-carrier family 2A3 (SLC2A3), high mobility group box 1 (HMGB1), signal transducer, and activator of transcription 3 (STAT3), metallothionein 3 (MT3), hypermethylated in cancer-1 (HIC1), and arachidonate 5-lipoxygenases (ALOX5) showed a significant relationship with overall survival time or overall survival state in patients with LUSC (Figure 3). Of these genes, DUSP1, ZFN36, and ALOX5 methylation status also have a significant relationship with pathological *M*, and ALOX5 methylation is significantly related to pathological *N* (Figures 3(a), 3(b) and 3(h)).

**Figure 4:**
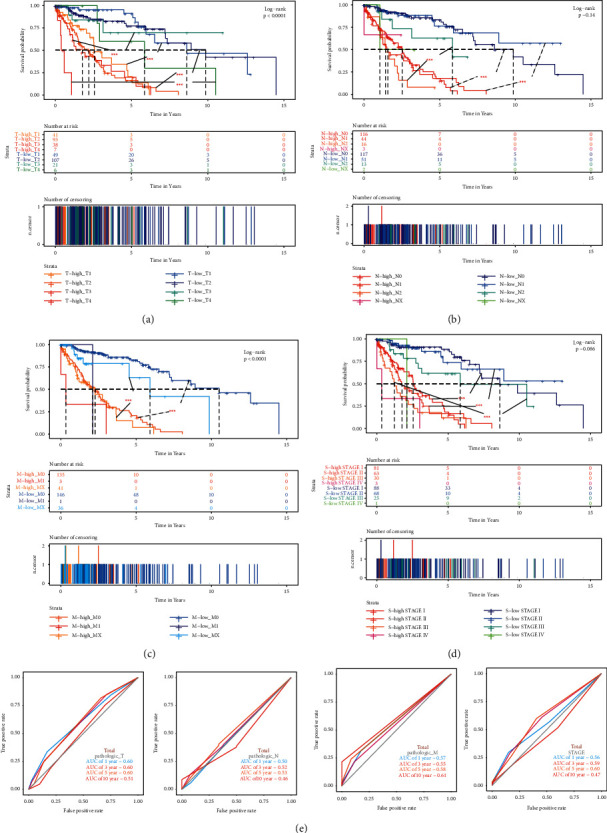
Kaplan–Meier survival analysis for OS in the high-risk and low-risk group compared with different clinical indicators of (a) subgroups with T1, T2, T3, and T4. (b) Subgroups with N0, N1, N2, and Nx. (c) Subgroups stratified by M0, M1, and MX. (d) Subgroups are stratified by stage I stage II, stage III, and stage IV. (e) ROC curve analysis of T, N, M, and stage according to the 1-, 3-, 5-, and 10-year survival of the area under the AUC value in the total TCGA cohort.

By the analysis of the TCGA total cohort, the high-risk group showed a shorter survival time than low-risk groups in the LUSC patients with the same *T*, *N*, *M*, and stage (Figures 4(a)–4(d)). *T*, *N*, *M*, and stage have long been recognized as prognostic predictive factors in LUSC patients. However, the prognostic prediction efficiency of these clinicopathologic features was lower than that of the DNA methylation signature. The AUC for 1-, 3-, 5-, and 10-year OS of *T* was 0.60, 0.60, 0.60, and 0.51 (Figure 4(e)) compared to the AUC of DNA methylation signature in the total cohort (0.80, 0.82, 0.87, and 0.94) (Figure 2(f)).

### 3.4. Identification of FRGs Signature Associated with Prognosis

**Figure 5:**
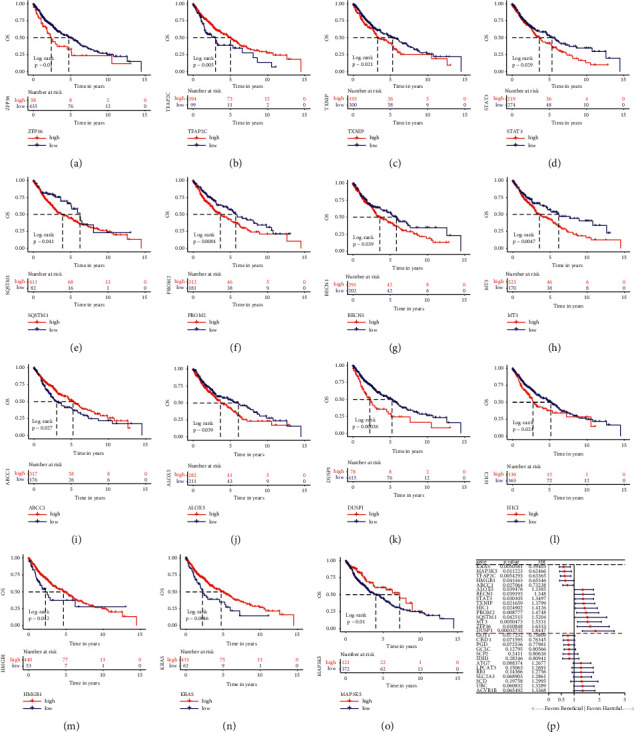
Kaplan–Meier survival analysis showed that among these signature-related genes, the expression level of (a) ZFP36, (b) TFAP2C, (c) TXNIP, (d) STAT3, (e) SQSTM1, (f) PROM2, (g) BECN1, (h) MT3, (i) ABCC1, (j) ALOX5, (k) DUSP1, (l) HIC1, (m) HMGB1, (n) KRAS, and (o) MAP3K5 showed a significant relationship with OS in LUSC patients. (p) The forest plot for the univariate Cox regression analysis results for the high and low expression group of signature-related genes.

Survival analysis was performed in LUSC patients to investigate the prognostic significance of FRGs by using the Kaplan–Meier method. The lower expression of a transcription factor activating protein 2 gamma (TFAP2C), multidrug resistance protein 1/MRP1 (ABCC1), HMGB1, V-Ki-ras2 Kirsten rat sarcoma viral oncogene homolog (KRAS), and mitogen-activated protein kinase kinase kinase 5 (MAP3K5) and the higher expression of zinc finger protein 36 (ZFP36), thioredoxin-interacting protein (TXNIP), signal transducer and activator of transcription 3 (STAT3), sequestosome 1 (SQSTM1), prominin 2 (PROM2), BECN1, MT3, ALOX5, DUSP1, and HIC1 were significantly correlated with poor overall survival in LUSC patients (Figures 5(a)–5(o)). Furthermore, the forest plot was used to show the hazard ratio (HR) for the high and low expression groups of each signature-related FRG (Figure 5(p)).

### 3.5. Genomic Alterations of FRGs in CESC

**Figure 6:**
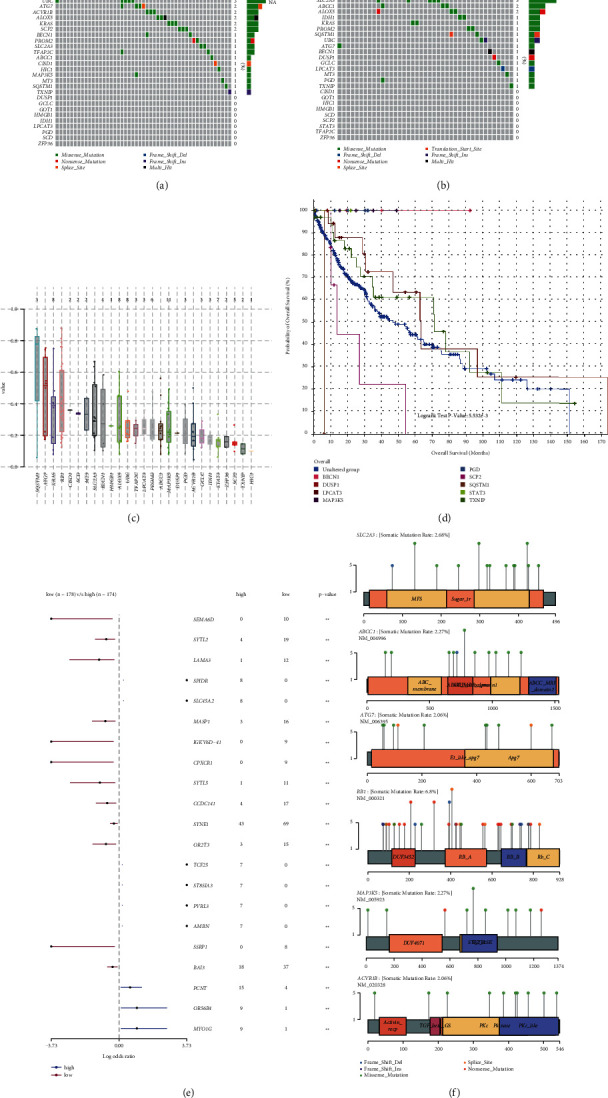
Mutation data analysis of signature-related genes in LUSC. Waterfall plots mutation results that show frequently mutated genes in the high-risk group (a) and the low-risk group (b). (c) The variant allele frequency (VAF) distribution of FRGs in LUSC by plotVaf function in “maftools” package. Each dot in the boxplot represents a variant. The total number of variants is listed on the top of each box. (d) Kaplan–Meier survival analysis showed mutations of some signature-related genes (BECN1, DUSP1, LPCAT3, MAP3K5, PGD, SCP2, SQSTM1, STAT3, and TXNIP) can be certified to be significantly associated with OS in LUSC patients compared with unaltered patients (*p* < 0.05). (e) The high-risk and low-risk group had 21 significantly different mutation frequencies. (f) Mutation type and domain of FRGs with a mutation rate exceeding 2%.

There were 27.84% of patients with mutations of these FRGs from genetic alterations analysis in LUSC patients, and the samples in the high-risk group own a significantly larger number of variants (28.16%) than those in the low-risk group (23.03%) (Figures 6(a) and 6(b)). The signature-related genes with mutation rate amounted to 19 genes containing RB1, SLC2A3, ABCC1, MAP3K5, ACVR1B, ATG7, KRAS, ALOX5, UBC, PROM2, STAT3, SCP2, BECN1, GCLC, IDH1, LPCAT3, PGD, SQSTM1, and TFAP2C, of which RB1 was found with the highest mutation rate in both high- and low-risk groups. The high-risk group contained a higher mutation rate in RB1, STAT3, UBC, ATG7, SCP2, KRAS, TFAP2C, and ABCC1, while a lower mutation rate was observed in ACVR1B, SLC2A3, ABCC1, MAP3K5, and so on. Among these mutations, the most common type is a missense mutation (Figures 6(a) and 6(b)). The variant allele frequency (VAF) distribution of FRGs in LUSC is shown in Figure 6(c). By Kaplan–Meier survival analysis, mutations of some signature-related genes (BECN1, DUSP1, LPCAT3, MAP3K5, PGD, SCP2, SQSTM1, STAT3, TXNIP) can be certified to be significantly associated with OS in LUSC patients compared with unaltered patients (*p* < 0.05) (Figure 6(d)). Interestingly, the two groups also had 21 significantly different mutation frequencies, as shown in Figure 6(e). In addition, there are a total of 6 FRGs with mutation rates exceeding 2%: RB1 (6.8%), SLC2A3 (2.68%), ABCC1 (2.27%), MAP3K5 (2.27%), ACVR1B (2.06%), and ATG7 (2.06%) (Figure 6(e)). Moreover, the data of concomitant occurrence among FRGs in NSCLC patients had no statistical significance (Figure 6(f)).

### 3.6. Function Enrichment Analysis and PPI Network of FRGs

**Figure 7:**
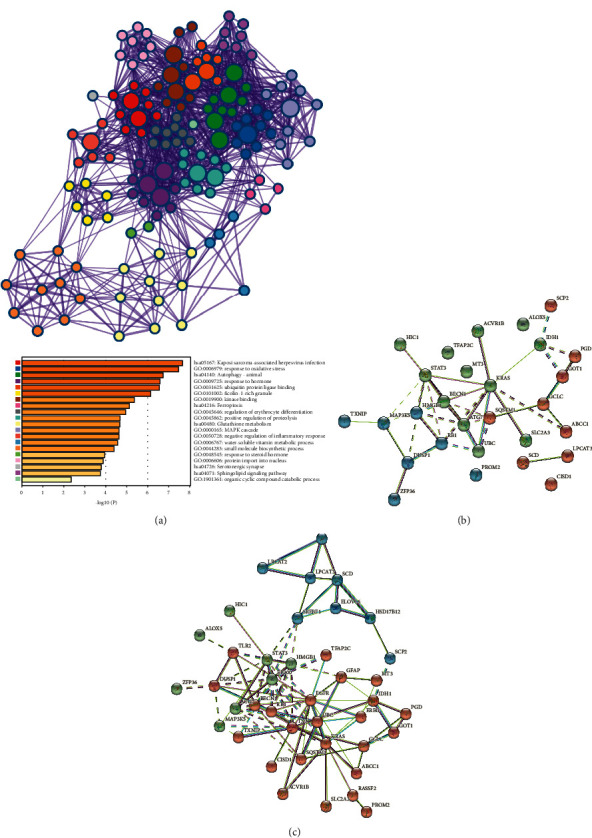
Function enrichment analysis and PPI network of FRGs. (a) GO and KEGG functional enrichment analysis by metascape revealed the biological processes and molecular functions for the 28 signature-related genes. (b) The PPI networks of 28 FRGs in LUSC. (c) Through the string database, the direct interact genes for TFAP2, MT3, ALOX5, SCD, LPCAT3, CISD1, and PROM2 were added to better understand the direct protein–protein interaction network of these signature-related genes.

Function enrichment analysis revealed that these 28 FRGs enriched in biological pathways were closely related to cancers, such as response to oxidative stress, apoptotic signaling pathway, cellular responses to external stimuli, regulation of cellular response to stress, autophagy-animal, ferroptosis, reactive oxygen species metabolic process, and response to oxygen levels (Figure 7(a)). Based on the STRING database, the PPI network of the 28 FRGs indicated that BECN1, ATG7, SQSTM1, HMGB1, and KRAS were the core genes of these FRGs (Figure 7(b)). To better understand the direct role relationship of these genes, we searched through the STRING database to find the direct interaction genes for TFAP2, MT3, ALOX5, SCD, LPCAT3, CISD1, and PROM2 that have no or a less direct relationship with other signature-related genes; the correlation between these FRGs and their interactive genes in LUSC is presented in Figure 7(c).

### 3.7. Immune Infiltration Analysis of FRGs

**Figure 8:**
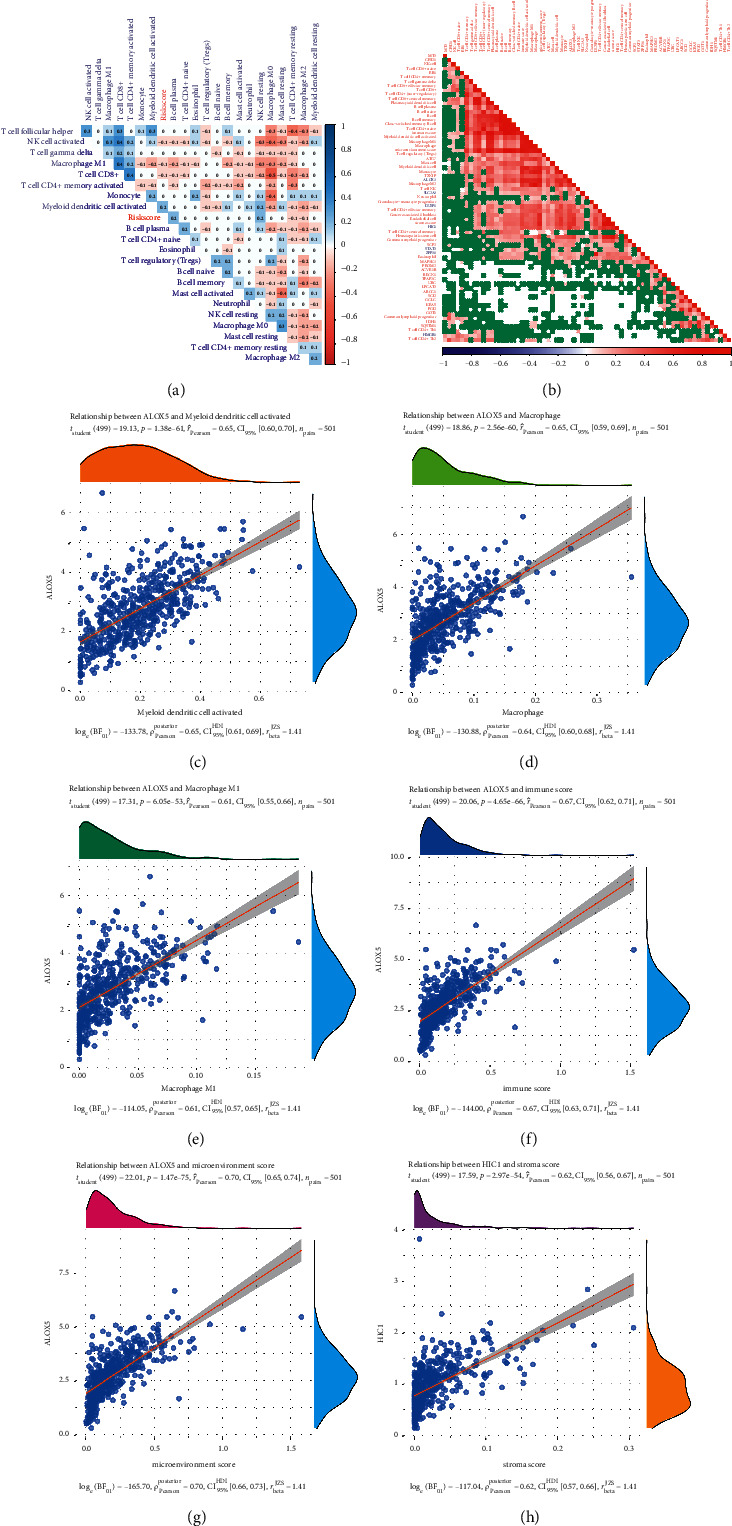
Immune infiltration analysis. (a) FRG-related risk score correlated with the proportions of immune cells. (b) The relationship between FRGs and immune infiltration types. (c)–(h) The relationship between FRGs and immune influence genes.

In addition, negative or positive correlation patterns were presented among the different infiltrating immune cells with risk scores or signature-related genes in patients with LUSC (Figure 8). FRGs-related risk score positively correlated with the proportions of *B*-cell plasma, resting NK cells, and activated myeloid dendritic cells, inversely correlated with resting CD4^+^*T*-cell memory, CD8^+^*T* cells, activated NK cells, and macrophages (Figure 8(a)). All these FRGs also showed a significant relationship with some immune infiltration types (Figure 8(b)). Among these significant immune influence genes, ALOX5, SLC2A3, DUSP1, and HIC1 showed the most positive correlations, while HMGB1, SQSTM1, GOT1, PGD, KRAS, GCLC, SCD, and ABCC1 showed the most negative correlations. The top 6 significant correlations are shown in (Figures 8(c)–8(h)).

### 3.8. Development and Validation of the Prognostic Nomogram Survival Model

**Figure 9:**
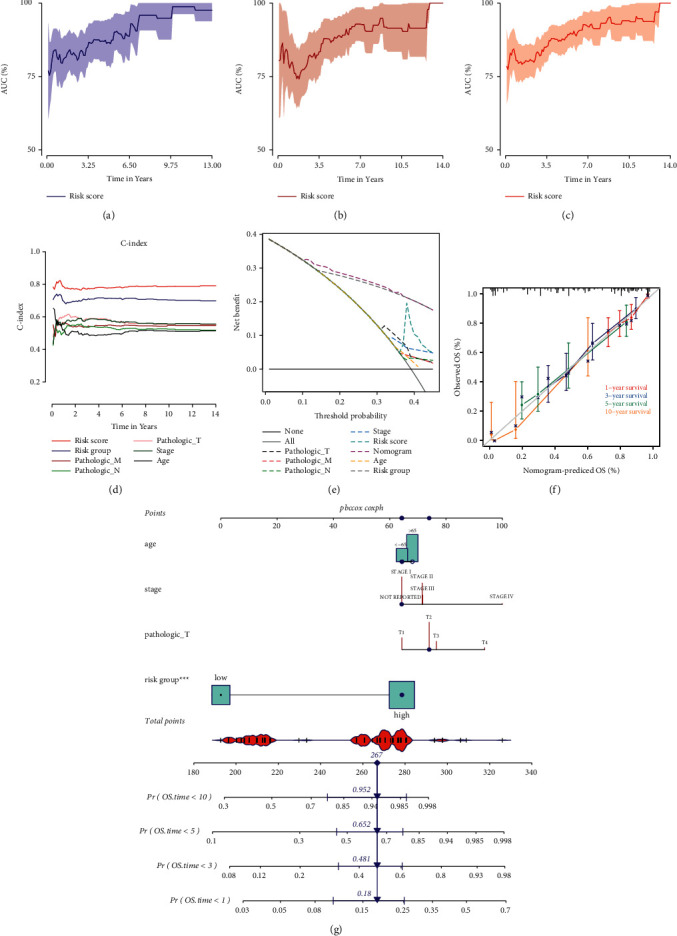
Nomogram construction and validation based on 31-DNA methylation signature. The AUC of 0–14 years OS predictions by risk score in the training (a), validating (b), and total cohort (c). (d, e) The *C*-index and DCA for comparing the risk score and risk group to these pathological statuses of LUSC. (f) Calibration plots of the nomogram. (g) The nomogram for predicting the proportion of patients with 1-, 3-, 5-, or 10-year OS.

We constructed the prognosis model containing 31 sites (cg24897291, cg11757894, cg00170343, cg06120945, cg18287222, cg01015199, cg18245652, cg05618386, cg17987505, cg27182551, cg13557397, cg25671164, cg18879829, cg22341865, cg17149920, cg17197538, cg00592510, cg15590007, cg00589914, cg20229027, cg05834353, cg00738178, cg07051257, cg08719701, cg23327734, cg12414653, cg03264601, cg06378498, cg05170326, cg10356455, and cg15871766). By Multivariate Cox regression analysis, DNA methylation sites-related risk score was an independent risk factor for the prognosis of LUSC patients (Supplementary Figure 3(a)). A LASSO regression analysis was established to select the prognosis factors of OS in LUSC patients (Supplementary Figures 3(b), 3(c)). According to the LASSO regression, a nomogram containing 4 factors including age, pathologic *T*, stage, and risk group was established for 1-, 3-,5-, and 10-year survival probability estimates (Figure 9(g)). As shown in Figures 9(a)–9(c), the AUC of 1-, 3-, 5-, and 10-year OS predictions are all above 0.75 in the training, validating, and total cohort. The *C*-index for the risk score and risk group is all above these pathological statuses of LUSC (Figure 9(d)). In Figure 9(f), the calibration curves showed an optimal accuracy between the nomogram predicted probability of 1-, 3-, 5-, and 10-year survival rates and actual data in LUSC patients. Meanwhile, the DCA results showed that the nomogram was supreme beneficial compared with other prognostic factors alone (Figure 9(e)). Moreover, the comparison of FRG DNA methylation signature with other 7 prognostic signatures of LUSC analyzed by survIDINRI showed that our model was significantly more effective than the 7 progonostic models of LUSC from published papers (Supplementary Figure 4).

## 4. Discussion

LUSC and lung adenocarcinoma (LUAD) are the two most common histological subtypes of NSCLC [37]. Some patients with LUAD could benefit from the molecular targeted therapy like tyrosine kinase inhibitors (TKIs) owing to the genetic heterogeneities such as epidermal growth factor receptor (EGFR) mutations, anaplastic lymphoma kinase (ALK) rearrangements, ROS1 fusions, and BRAF mutations [38]. Compared to LUAD, LUSC patients rarely has the mutation of EGFR and ALK rearrangements and no other effective driver genetic mutation, which postponed the progress of targeted drugs.

It is an enormous challenge to explore effective individualized therapy for patients with LUSC. Therefore, it is necessary to discover powerful prognostic biomarkers, which will contribute to developing novel and efficient therapeutic targets for LUSC. Ferroptosis is one form of regulated cell death (RCD) characterized by iron accumulation and lipid peroxidation and plays a significant role in cancer progression and treatment [7, 39]. The various FRGs signatures have been identified as predictive prognosis models in many cancers, such as glioma, breast cancer, lung adenocarcinoma, renal cell carcinoma, and hepatocellular carcinoma [11, 40–43]. In the latest research report, a prognostic model was constructed for NSCLC patients based on 21 FRGs [44]. And, the pathways enriched with differentially expressed FRGs were related significantly to immunosuppressive status [44]. There are enormous differences in gene-phenotype, therapeutic modalities, and prognosis between patients with LUAD and LUSC. Therefore, it is necessary to construct an exclusive prediction model based on FRGs to forecast the prognosis of the LUSC cohort. Although the evidence was limited, the analysis of TCGA data for LUSC showed that TP63 amplification could enhance the glutathione metabolism pathway involved in ferroptosis regulation [45]. However, our analysis indicated that the prognostic prediction efficiency of FRGs was not satisfactory for patients with LUSC. DNA methylation, as a major epigenetic alteration, has been implicated in the regulation of gene expression by DNA methyl-transferase (DNMT) [15]. In addition, the critical role of DNA methylation has been certified in cancer initiation and progression [46]. Several methylation biomarkers have been used to predict prognosis in LUSC [16–18]. The prognostic prediction model based on the DNA methylation site showed better prediction efficiency in LUSC [18, 47, 48]. However, the DNA methylation signature of FRGs has not been investigated in the prognostic prediction of LUSC. Based on the TCGA data, our study identified the genes and DNA methylation sites of FRGs associated with the OS in LUSC. The 31 methylation sites from 28 ferroptosis-related genes were revealed to be tightly correlated with the prognosis of LUSC patients. The risk prognosis model based on 31 methylation sites of FRGs was superior to the model based on clinicopathologic features in the prediction of 1-, 3-, 5-, and 10-year OS in LUSC. In addition, the accuracy and efficiency of this risk prognosis model were significantly superior to other published models [17, 18, 27–31] for LUSC patients.

Among 28 FRGs, the overall DNA methylation status of DUSP1, ZFP36, SLC2A3, HMGB1, STAT3, MT3, HIC1, and ALOX5 effectively influences the OS of patients with LUSC. DUSP1, as a protein phosphatase, abnormally expressed in various cancers, played a key role in tumor immunotherapy, and was associated with prognosis [49]. The deficit of DUSP1 in bone marrow-derived macrophages evaluated the production of TNF-a and IL-10 by ZFP36 phosphorylation, which leads to the immunomodulation functions [50, 51]. High expression of SLC2A3, also known as glucose transporter 3(GLUT3), portends a poor prognosis of the patients with most cancer types including lung squamous cell carcinoma [52]. SLC2A3 was also implicated in the immunoregulatory effects [53]. Cytoplasmic HMGB1 could mediate antitumor immunity through regulating immunogenic cell activity [54]. Targeting STAT3 had been highlighted as an effective therapeutic approach in the regulation of tumorigenesis and immune escape [55]. MT3 was a small cysteine-rich protein that played an important role in tumor growth and immune escape [56]. HIC1 played as a tumor suppressor by hypermethylation mediated function loss [57]. A leukotriene-generating enzyme ALOX5 had been identified as a novel ELF3 target gene that is implicated in the immune response modulation [58]. In brief, DUSP1, ZFP36, SLC2A3, HMGB1, STAT3, MT3, and ALOX5 were all FRGs that might be involved in cancer development and immune regulation.

Most noteworthy, ferroptosis plays an emerging role in the regulation of antitumor immunity through influencing immune cells, tumor microenvironment (TME), and the crosstalk between immune cells and tumor cells [59]. Immune escape is a major contributing factor to the malignant progression of cancer, which was regulated by the disturbance of cell immune function (e.g., decreased immunological activated cells and immunosuppressive cells), and overexpression of immune checkpoint genes including programmed cell death-1 (PD-1), cytotoxic T lymphocyte antigen 4 (CTLA-4), and lymphocyte-activation gene 3 (LAG3) in TME [60, 61]. Immune checkpoint blockade was already widely used as the most popular anticancer immunotherapy for patients with the advanced driver gene negative NSCLC and could induce tumor cell ferroptosis through the activation of CD8^+^*T* cells [62]. Interestingly, ferroptosis might affect various immune cells resulting in the promotion of tumor immune evasion [59]. Importantly, the inhibition of ferroptosis in CD8^+^ T cells not only restored antitumor efficacy effectively but also enhanced the antitumor activity of PD-1 blockade combination [63]. NSCLC patients with features of mutations in the immune system may benefit from immunotherapy. Despite the success of immune checkpoint inhibitors for LUSC, only a small portion of patients exerts an effective immune response, which leads to cancer suppression.

Analysis of tumor-infiltrating lymphocytes (TILs) showed that more CD4, CD8, neutrophil, macrophages, and dendritic cells were detected in high prognostic risk LUSC patients compared to low prognostic risk LUSC patients [64]. Our result also revealed that FRGs-related risk score was significantly correlated with TILs in patients with LUSC, such as B, NK, dendritic, CD4^+^, CD8^+^ cells, and macrophages. CD4^+^, CD8^+^, and NK cells, as cytotoxic lymphocytes (CTL), can eliminate cancer cells resulting in the inhibition of cancer development [65]. The high FRG-related risk score we constructed negatively correlated with the proportions of CD4^+^, CD8^+^ T cells, and NK cells in patients with LUSC, which might be a valuable contribution to the poor prognosis of high-risk patients.

## 5. Conclusion

In summary, we studied the prognostic value of ferroptosis-related epigenetic features such as DNA methylation in LUSC. We screened ferroptosis-related genes from the FerrDb database and the methylation data of these related genes in LUSC methylation data from the TCGA database. The FRG DNA methylation data-based prognostic model was conducted to act as a powerful prognostic prediction indicator in LUSC patients and is advantageous over the traditional model based on *T*, *N*, *M*, and stage.

## Data Availability

The original contributions presented in the study are included in the article/Supplementary Materials, further inquiries can be directed to the corresponding authors.

## Supplementary Materials

Supplementary Materials Supplementary Table 1 and Supplementary Figures 1–4 are found in “Supplementary Materials.” Supplementary Table 1. The clinical features comparation between the training set and the validating set. Supplementary Figure 1. Characterizing a large methylation heterogeneity in LUSC tumor tissues. Methylation levels density and multidimensional scaling (MDS) showed tumor samples own high heterogeneity compared with normal samples in both the total samples and paired samples. (a, d) The methylation density plot in paired and total samples; (b, e) the MDS plots of methylation level in paired and total samples. (c, f) Sample methylation level clustering in paired and total LUSC samples. (g, h) The PCA on the methylation levels of 4365 FGR methylation sites in paired and total samples. Supplementary Figure 2. Identification of DNA methylation signature of FRGs associated with prognosis. (a) Univariate Cox proportional hazards regression identified 137 prognostic DNA methylation sites of FRGs. (b, c) DNA methylation signature constructed by LASSO regression related to OS with nonzero coefficients. (d) The cutoff point of the risk score is derived from the “Surv_cutpoint” function in “survminer.” Supplementary Figure 3. The risk group is a prognosis factor for LUSC. (a) Multivariate Cox proportional hazards regression showed that risk group is an independent prognosis factor. (b, c) DNA methylation signature constructed by LASSO related to OS with nonzero coefficients. (d) LASSO regression identified the prognosis factors for the nomogram. Supplementary Figure 4. Clinical benefit evaluation by NRI and NRI analysis. “survIDINRI” package was used to analysis the IDI, continuous NRI, and median improvement of LUSC FRG signature compared with other seven published LUSC prediction signatures. Empirical distribution function of the change in estimated risk score for *T*0 ≤ *t*0 (thick solid line) and *T*0 > *t*0 (thin solid line). The difference between areas under two curves is IDI and corresponding (1-alpha/2) confidence interval. The distance between two black dots is continuous -NRI and corresponding (1-alpha/2) confidence. The distance between two gray dots is median improvement in risk score and corresponding (1-alpha/2) confidence.
