# Supplementary material for: A Novel Prognosis Signature Based on Ferroptosis-Related Gene DNA Methylation Data for Lung Squamous Cell Carcinoma
Source: J Oncol. 2022 Sep 12;2022:9103259. doi: 10.1155/2022/9103259 (PMC9484906; doi:10.1155/2022/9103259)
Supplement: Supplementary Materials — Supplementary Table 1 and Supplementary Figures 1–4 are found in “Supplementary Materials.” Supplementary Table 1. The clinical features comparation between the training set and the validating set. Supplementary Figure 1. Characterizing a large methylation heterogeneity in LUSC tumor tissues. Methylation levels density and multidimensional scaling (MDS) showed tumor samples own high heterogeneity compared with normal samples in both the total samples and paired samples. (a, d) The methylation density plot in paired and total samples; (b, e) the MDS plots of methylation level in paired and total samples. (c, f) Sample methylation level clustering in paired and total LUSC samples. (g, h) The PCA on the methylation levels of 4365 FGR methylation sites in paired and total samples. Supplementary Figure 2. Identification of DNA methylation signature of FRGs associated with prognosis. (a) Univariate Cox proportional hazards regression identified 137 prognostic DNA methylation sites of FRGs. (b, c) DNA methylation signature constructed by LASSO regression related to OS with nonzero coefficients. (d) The cutoff point of the risk score is derived from the “Surv_cutpoint” function in “survminer.” Supplementary Figure 3. The risk group is a prognosis factor for LUSC. (a) Multivariate Cox proportional hazards regression showed that risk group is an independent prognosis factor. (b, c) DNA methylation signature constructed by LASSO related to OS with nonzero coefficients. (d) LASSO regression identified the prognosis factors for the nomogram. Supplementary Figure 4. Clinical benefit evaluation by NRI and NRI analysis. “survIDINRI” package was used to analysis the IDI, continuous NRI, and median improvement of LUSC FRG signature compared with other seven published LUSC prediction signatures. Empirical distribution function of the change in estimated risk score for T0 ≤ t0 (thick solid line) and T0 > t0 (thin solid line). The difference between areas under two curves is IDI and cor [file Supplementary Figure 1.pdf › 9103259.f1/Supplementary Figure 1.pdf]

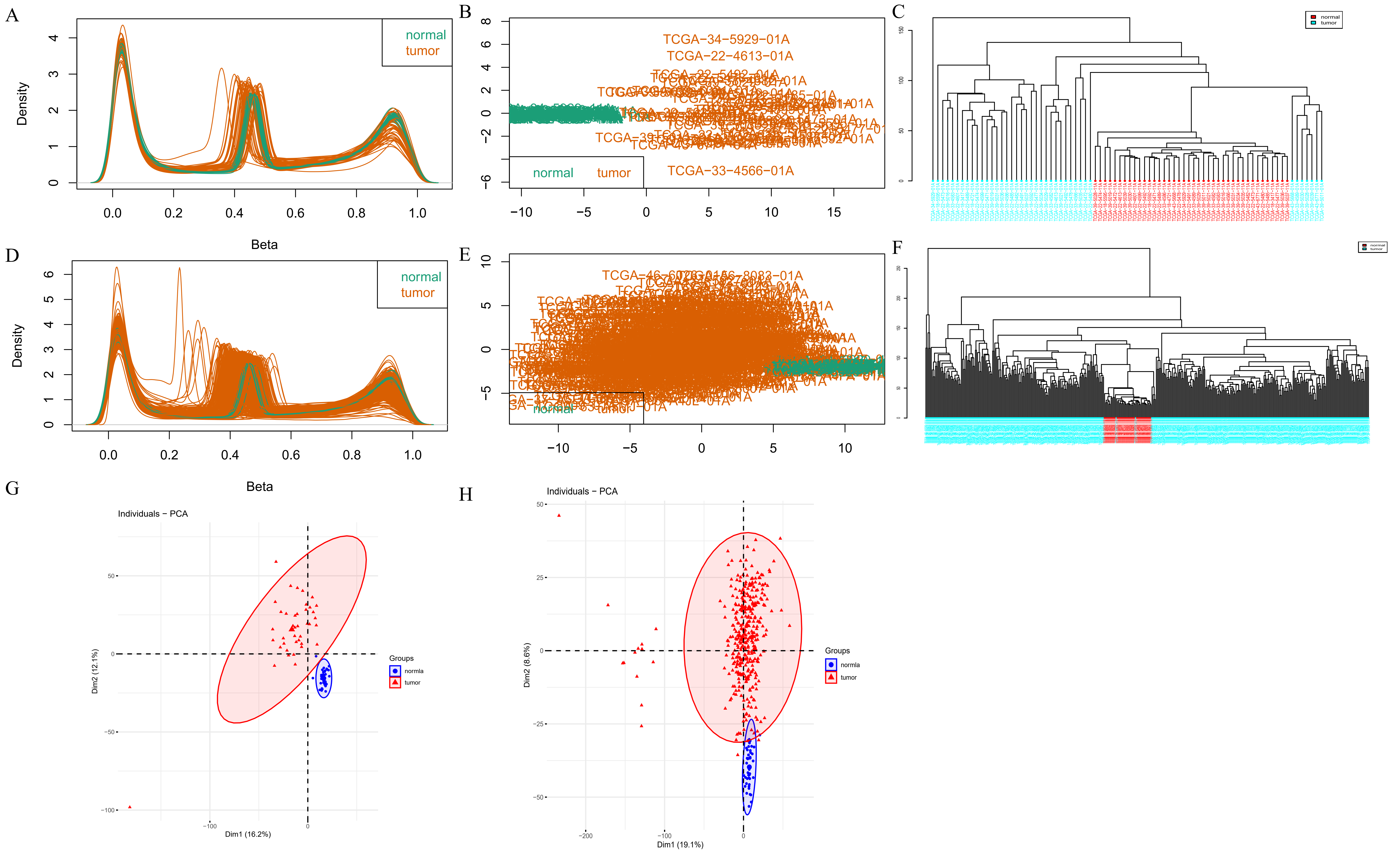

A
Density
Beta
normal
tumor
B
TCGA−34−5929−01A
TCGA−22−4613−01A
TCGA−33−4566−01A
normal
tumor
C
normal
tumor
D
Density
Beta
normal
tumor
E
normal
tumor
F
normal
tumor
G
Individuals − PCA
Dim1 (16.2%)
Dim2 (12.1%)
Groups
normla
tumor
H
Individuals − PCA
Dim1 (19.1%)
Dim2 (8.6%)
Groups
normla
tumor
